# Developing the Total Health Profile, a Generalizable Unified Set of Multimorbidity Risk Scores Derived From Machine Learning for Broad Patient Populations: Retrospective Cohort Study

**DOI:** 10.2196/32900

**Published:** 2021-11-26

**Authors:** Abhishaike Mahajan, Andrew Deonarine, Axel Bernal, Genevieve Lyons, Beau Norgeot

**Affiliations:** 1 Anthem Inc Palo Alto, CA United States; 2 XY Health Cambridge, MA United States

**Keywords:** multimorbidity, clinical risk score, outcome research, machine learning, electronic health record, clinical informatics, morbidity, risk, outcome, population data, diagnostic, demographic, decision making, cohort, prediction

## Abstract

**Background:**

Multimorbidity clinical risk scores allow clinicians to quickly assess their patients' health for decision making, often for recommendation to care management programs. However, these scores are limited by several issues: existing multimorbidity scores (1) are generally limited to one data group (eg, diagnoses, labs) and may be missing vital information, (2) are usually limited to specific demographic groups (eg, age), and (3) do not formally provide any granularity in the form of more nuanced multimorbidity risk scores to direct clinician attention.

**Objective:**

Using diagnosis, lab, prescription, procedure, and demographic data from electronic health records (EHRs), we developed a physiologically diverse and generalizable set of multimorbidity risk scores.

**Methods:**

Using EHR data from a nationwide cohort of patients, we developed the total health profile, a set of six integrated risk scores reflecting five distinct organ systems and overall health. We selected the occurrence of an inpatient hospital visitation over a 2-year follow-up window, attributable to specific organ systems, as our risk endpoint. Using a physician-curated set of features, we trained six machine learning models on 794,294 patients to predict the calibrated probability of the aforementioned endpoint, producing risk scores for heart, lung, neuro, kidney, and digestive functions and a sixth score for combined risk. We evaluated the scores using a held-out test cohort of 198,574 patients.

**Results:**

Study patients closely matched national census averages, with a median age of 41 years, a median income of $66,829, and racial averages by zip code of 73.8% White, 5.9% Asian, and 11.9% African American. All models were well calibrated and demonstrated strong performance with areas under the receiver operating curve (AUROCs) of 0.83 for the total health score (THS), 0.89 for heart, 0.86 for lung, 0.84 for neuro, 0.90 for kidney, and 0.83 for digestive functions. There was consistent performance of this scoring system across sexes, diverse patient ages, and zip code income levels. Each model learned to generate predictions by focusing on appropriate clinically relevant patient features, such as heart-related hospitalizations and chronic hypertension diagnosis for the heart model. The THS outperformed the other commonly used multimorbidity scoring systems, specifically the Charlson Comorbidity Index (CCI) and the Elixhauser Comorbidity Index (ECI) overall (AUROCs: THS=0.823, CCI=0.735, ECI=0.649) as well as for every age, sex, and income bracket. Performance improvements were most pronounced for middle-aged and lower-income subgroups. Ablation tests using only diagnosis, prescription, social determinants of health, and lab feature groups, while retaining procedure-related features, showed that the combination of feature groups has the best predictive performance, though only marginally better than the diagnosis-only model on at-risk groups.

**Conclusions:**

Massive retrospective EHR data sets have made it possible to use machine learning to build practical multimorbidity risk scores that are highly predictive, personalizable, intuitive to explain, and generalizable across diverse patient populations.

## Introduction

Multimorbidity risk scores, which factor in the presence of several chronic conditions, can provide insights into morbidity and mortality [1,2]. In general, the number of co-occurring medical conditions is associated with increased adverse medical outcomes [3-5] and increased use of medical services [6]. This is particularly true for older individuals since the number of co-occurring medical conditions will increase with age [7]. Various approaches to quantifying multimorbidity have been used, including simply counting the number of conditions [8], while more complex tools have also been developed, such as the Charlson Comorbidity Index (CCI) [9] and the Elixhauser Comorbidity Index (ECI) [10]. However, these scores were developed decades ago and are limited to diagnostic information or certain populations, which may limit their utility. A systematic review [11] of multimorbidity scores examined 35 major multimorbidity scores, which could be subclassified by the information they used (eg, prescription data, diagnostic data, self-reported quality of life) and the outcomes they recorded (all-cause mortality, emergency room admissions, and hospital admissions). Patients with multimorbidities are cared for in general practice and by specialists [8,12,13] who use disease-specific risk scores and guidelines. Condition-specific risk scores, such as the Framingham Risk Score [14] for coronary heart disease, can help identify specific interventions to benefit patients and provide actionable information to guide tests and medications. One potential issue with the use of these tools is that sometimes they do not address the overall health care priorities of the patient due to their narrow focus [8,15,16].

Multimorbidity scores tend to only use one type of clinical data, such as diagnoses, prescriptions, or procedures, and rarely integrate them. As a result, they may be missing vital information and relationships in patient information. Although newer methods, such as probabilistic phenotyping [17], may alleviate these concerns, while remaining scalable, these methods are still highly experimental, with a wide variety of methods and little consensus on which ones are most trustable for real-world settings [18]. Using multiple data sets, feature types, and methodological explorations could provide a more comprehensive and robust estimate of multimorbidity risk. Currently, no multimorbidity scores exist that produce granular and overall risk profiles irrespective of age and sex; are derived from a large, representative population of patients; and integrate multiple clinical data sets, including diagnoses, prescriptions, lab results, and procedures using machine learning (ML; building upon previous ML-based strategies and recommendations for multimorbidity analysis by Hassaine et al [19]). Such scores could help health care providers engage in patient-centered care and prescribing, reduce polypharmacy, and guide deprescribing when used together with traditional risk scores and guidelines.

To address this need, we sought to create the total health profile (THP), a set of ML-derived measures of an individual's comprehensive clinical risk. The THP presents clinical risk in five separate models (referred to as “component scores”), producing granular, multimorbid risk scores specific to cardiovascular (“heart score”), respiratory (“lung score”), neuropsychiatric (“neuro score”), renal (“kidney score”), and gastrointestinal (“digestive score”) conditions. These organ systems reflect those involved in the top 10 sources of mortality in the United States [20] and serve to complement existing disease-specific risk scores. We also included, as a sixth score, the total health score (THS), a single view of a patient's overall health across all five of the aforementioned organ systems, which can be compared to existing pure multimorbidity risk scores. Each of these six scores was independently modeled using electronic health record (EHR) data consisting of demographic information, diagnosis codes, lab results, prescriptions, and medical procedural data and required, otherwise, no patient behavior or familial history data. For the unified risk endpoint of all six of the scoring models, we used inpatient (IP) hospital visits. As such, each score’s estimate of clinical risk represents the likelihood of an IP hospital visit over the next 24 months, attributable to the score's clinical category (eg, lung, heart). After training, testing, and calibrating the THS and the five organ system component scores, we analyzed the metrics and generalizability of each score across populations. We also conducted ablation tests of several feature groups to assess their importance in the final set of models. Finally, we discussed the clinical applicability of the THP, limitations of the study, and future work.

## Methods

### Study Design and Patient Inclusion Criteria

This retrospective cohort study used lab measurements and an administrative claims database of 52 million patients provided by a US health care insurance company. Patients were enrolled in a mixture of commercial, Medicare, Medicaid, and exchange plans. Our study design involved training on retrospective data from a certain time window and assessing performance via a follow-up time window. The retrospective observation period, or the time period in which model features were collected, was defined as January 1, 2016, through December 1, 2017, and the follow-up period, or the time period in which the model labels were collected, as January 1, 2018, through December 31, 2019.

All patient data were de-identified. Patients selected for inclusion had at least one medical claim in each year of the data collection and follow-up periods and had a known sex, birthdate, and zip code. These inclusion criteria resulted in 14 million patients, from which 1 million patients were randomly selected for analysis using PySpark, resulting in 992,868 patients due to the approximation methods used by PySpark. This patient sample was split into training (n=794,294) and testing (n=198,574) groups corresponding to an 80:20 ratio. Diagnosis codes (*International Classification of Diseases, Tenth Revision* [ICD-10]), medical procedure codes (Current Procedural Terminology [CPT]), lab data, demographics (social determinants of health [SDoH], patient gender/age), and prescription data (defined by General Product Identifier [GPI] codes) were used from patients who met the selection criteria. Our study, in total, used 88 ICD-10 codes and 30 chronic conditions (derived from ICD-10 codes specified by the ECI), 16 lab types, 764 GPI codes representing 4 GPI prefixes, 14 CPT codes, and 17 demographic markers.

### Data Processing

Using the data compiled for the 992,868 patients, we extracted a set of features corresponding to chronic diagnoses, acute diagnoses, IP hospital visits, prescriptions, sociodemographic information, and lab results/physical exam measurements for feature extraction and modeling. A description of all features gathered during the data collection period follows next.

Demographic information was extracted from the United States Census American Community Survey for 2017 at the zip code level. This information included population, household count, and race and ethnicity percentages for that zip code (eg, African American, non-Hispanic White, Hispanic, Asian, Native American), sex percentages per zip code, and economic indicators, including the mean and median income. Demographic data also included the age and sex of the patients. Chronic disease diagnoses were counted as the presence of a chronic disease, while acute diagnoses were counted as the number of those diagnoses in the study period, summed over the component. For instance, 3 atrial fibrillation codes and 2 acute heart failure codes during the 2-year data collection period would have resulted in the number of acute heart diagnoses being 5. Medical procedure features were counted as the count of IP CPT codes that occurred during the data collection period, with otherwise identical score-specific inclusion criteria to the IP hospital labels (discussed in the Model Outcome Labels section). Four groups of prescriptions were included, assigned using the first two digits of the GPI code and indicated by binary presence: antihypertensives, hypoglycemics, lipid-lowering medications, and antithrombotic agents. In all, 16 labs or vitals were included in the study, each one being a numerical feature. If there were multiple results of the same lab data/vitals collected during the data collection period, only the most recent measurement was included. In total, our feature set and labels used the following set of clinical features: diagnoses (88 ICD-10 codes and 30 chronic conditions), labs (16 types), prescriptions (764 GPI codes, representing 4 GPI prefixes), procedures (14 CPT codes), and demographics (15 SDoH and 2 individual patient characteristics).

Except for demographic features, lab values, and vital signs, all input features were filtered on a model-by-model basis to include only score-relevant data (ie, the heart score would be modeled using only physician-curated features related to cardiovascular health). For IP hospital visit features collected during the data collection period, only score-specific IP visit counts were included (ie, the heart score would have as input the number of heart-related IP visitations during the data collection period, not the lung-related, and so on). The set of input features used over all component score models were used as input for the THS model, with an exception for chronic diagnosis features.

### Model Outcome Labels

All component score labels were a binary indicator referring to whether a patient had an IP visit within the follow-up period, given that they also had acute or chronic diagnoses within 12 months prior to the IP visit and within 7 days after the IP visit, establishing both a history of that condition and that the IP visit was (likely) related to that condition. These diagnoses would be specific to each component, given by the corresponding ECI comorbidities and ICD-10 codes. For example, a possible positive label for the lung scoring model could be an IP hospital stay CPT code on June 2, 2019, a diagnosis code corresponding to pneumonia 3 months prior to it, and a diagnosis code corresponding to chronic pulmonary disease 2 days after it. The THS label is simply the combination of all the component score labels; if a patient has any positive component score label, the THS label would be positive as well.

### Modeling Procedures and Baselines

All scores were calculated using a gradient-boosted tree classifier, with default hyperparameters, using the Scikit-Learn Python 3.6 package (version 0.24.1). Using demographics, diagnoses, lab values, procedures, and prescription data as input and IP visits as binary labels, separate models were trained for each score and subsequently calibrated using an isotonic regression with 3-fold cross-validation over the training set. Discriminative results from the models were obtained using the optimal threshold point of the training set (given by the threshold that yielded the smallest difference between the true-positive rate and the false-positive-rate) and applied to the testing set. All missing values were mean-imputed, and all input features for each model were mean-normalized using the training data.

We had multiple baselines: a logistic regression model with default hyperparameters using the *statsmodel* package (version 0.12.0) with otherwise identical feature sets, and a comparison of the performance of the THS to commonly used scores of a similar nature, specifically the CCI and the ECI, in predicting the hospitalization endpoint. We also conducted multiple ablation tests on the feature groups: a set of gradient-boosted tree classifiers, all with procedural data, but having only one set of either lab, SDoH, prescription, or diagnosis information. For the baseline gradient-boosted comparison with the combined feature model for patient subgroups, CI calculations were generated using 100 bootstrap iterations of 10% of the given demographic. The patient subgroups analyzed were patients with two or more of any comorbidity and one or more prescriptions of hypertensive, hyperglycemic, lipid-lowering, or antithrombotic medications.

### Radial Plots

Radial plots were generated using three patients who were closest to each of the centroids of a fitted, randomly initialized K-means model, with a K value of 3. The K-means algorithm used the Scikit-Learn Python 3.6 package (version 0.24.1), and the plots themselves were generated using Plotly.

### Model Discrimination and Generalization/Sensitivity Analyses

**Figure 1:**
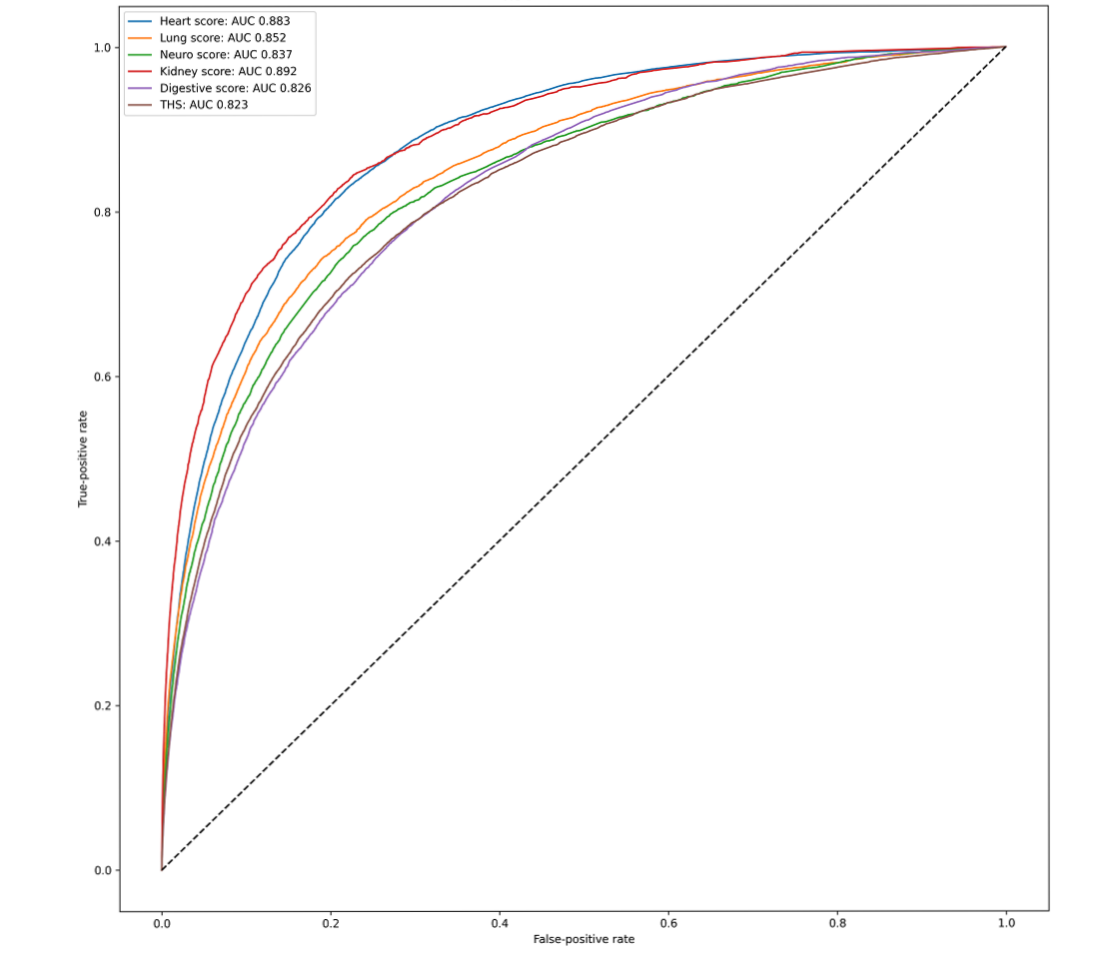
ROCs for all scores in the THP. AUC: area under the curve; ROC: receiver operating curve; THP: total health profile; THS: total health score.

Models were assessed on three levels: discriminative performance, calibration, and generalizability in performance across different demographics. To assess the discriminative performance of each model in the THP, we calculated the area under the receiver operating curve (AUROC), sensitivity, and specificity using Scikit-Learn for the testing set of 198,574 patients. We also plotted the AUROC for all scores on the testing set (Figure 1). All CIs for the discriminative metrics were generated using 500 bootstrap samples of 20,000 from the testing data set. We selected the AUROC as our primary metric because it represents a comprehensive measure of the true-positive-rate and false-positive-rate trade-off without needing an optimal threshold point. Since our outcomes exhibited strong class imbalance, which may have led to overly optimistic AUROC values, we used sensitivity and specificity as secondary model measures. To assess calibration performance, we created calibration plots using Scikit-Learn, graphing predicted probability versus positive label percentage across 10 uniform probability bins. We assessed calibration, as measured by calibration plots, as the primary measurement of clinical utility as it gives a clear idea of how these scores can be used to identify sick patients, avoid alarm fatigue, and be interpreted as a probabilistic likelihood. To assess the generalization performance of each model, we studied how the performance and scores of the models vary across age, zip code income, and sex categories. We plotted how the AUROC varies among age groups (decade age groupings), median income groups (low, medium and high), and binary gender (male or female). Additionally, we computed statistical significance Z-tests for AUROC pairwise differences between all groups within each category. Due to the lower sample sizes of the groups, CI calculations were generated using 100 bootstrap samples of 10% of the given demographic of the testing group. The THS and component scores were then analyzed by plotting the distribution of scores as a function of age and disease burden (measured by the presence of pre-existing comorbidities during the observation period). Specifically, we looked at the distributions of the THS and the component scores among various age groups for patients with zero comorbidities found during the data collection period and patients with at least one ECI comorbidity related to the given component found during the data collection period.

### Physician-Guided Feature Selection and Curation

To select features to incorporate into the THS, a physician-guided curation method was incorporated, which involved selecting conditions, reviewing clinical practice guidelines for important conditions, and identifying clinical measures, tests, and pharmacological interventions in those guidelines. No statistical feature selection techniques were used, as those offer improved accuracy mainly in cases with relatively small training data sets or models that are sensitive to unsuspected feature correlations [21].

An overview of the manually guided feature selection process is described below:

1. Selection of disease categories/subscores: The top causes of death across the United States were reviewed from the 2018 mortality statistics from the National Center for Health Statistics, and five main categories that the causes of death could be classified into were identified: cardiac (heart), respiratory (lung), neuropsychiatric (neuro), gastrointestinal (digestive), and renal (kidney).
2. We then obtained the leading causes of IP conditions for 2011-2013 using the Agency for Health care Research and Quality (AHRQ) Healthcare Cost and Utilization Project (HCUP) database. We cross-referenced the top 30 codes for each year with the 5 categories we developed in step 1. We did not include codes corresponding to obstetrical conditions, complications related to birth and delivery, multisystem malignancy, and musculoskeletal conditions.
3. Using the AHRQ HCUP codes, we extracted the ICD-10 codes and selected additional medically related conditions (eg, selecting the ICD-10 codes for ischemic stroke codes, in addition to ICD-10 codes for hemorrhagic stroke).
4. To obtain corresponding prescription, lab, and procedure codes, we then reviewed clinical practice guidelines for the identified conditions (eg, stroke, chronic obstructive pulmonary disease) from the United States Preventive Services Task Force [18,22]. These guidelines were then reviewed and lab data and procedures corresponding to diagnosis and management were extracted by a physician. These were then manually mapped to the corresponding drug (GPI codes), procedure (CPT codes), and lab (Logical Observation Identifiers Names and Codes) codes.

### MI-CLAIM Checklist

This work meets the Minimum Information about Clinical Artificial Intelligence Modeling (MI-CLAIM) requirements for sharing design, data/optimization, model performance, model examination, and level of reproducibility [23].

### Role of the Funding Source

The funding source collected the raw data.

### Ethics Approval

The analysis presented here is not to be characterized as human subject research. We are presenting the results of an analysis conducted for a health plan’s health care operations in accordance with the Health Insurance Portability and Accountability Act of 1996 (HIPAA). Only aggregated results of the business analysis are provided, and no individually identifiable information (protected health information or otherwise) was used in the development of this presentation.

## Results

### Patient Cohort

In all, 992,868 patients matched the inclusion criteria (Table 1). The majority were female (n=560,165, 56.4%), with 432,703 (43.6%) males, concordant with the 2019 census results [20]; the median age (41 years) was slightly higher than the national average; and the number of comorbidities tended to increase with age, consistent with previous findings [7]. The mean patient age was 39.07 years (95% CI 39.02-39.12), the mean number of comorbidities was 1.71 (95% CI 1.71-1.72), and the percentage of patients with IP visits was 1.65% (females 1.7%, males 1.7%). IP visits were also positively correlated with age for each organ system (Spearman correlation=0.314), which is concordant with previous studies [21]. The sample-weighted summary of zip-code-level demographics had a median income of $66,829, and the racial averages by zip code were 73.8% White, 5.9% Asian, and 11.9% African American, closely matching census averages (Table 1).

**Table 1:** Demographic profile of patients included in the analysis cohort (N=992,868).

Characteristics		Overall	Females	Males
**Age range, n (%)**				
	0-10 years	150,685 (15.2)	73,224 (48.6)	77,461 (51.4)
	10-20 years	140,684 (14.2)	73,657 (52.4)	67,027 (47.6)
	20-30 years	80,136 (8.1)	55,013 (68.6)	25,123 (31.4)
	30-40 years	102,397 (10.3)	65,797 (64.3)	36,600 (35.7)
	40-50 years	126,923 (12.8)	75,151 (59.2)	51,772 (40.8)
	50-60 years	163,675 (16.5)	91,305 (55.8)	72,370 (44.2)
	60-70 years	122,243 (12.3)	66,270 (54.2)	55,973 (45.8)
	70-80 years	72,560 (7.3)	39,633 (54.6)	32,927 (45.4)
	80-90 years	33,565 (3.4)	20,115 (59.9)	13,450 (40.1)
**Number of comorbidities, n (%)**				
	0	362,469 (36.5)	202,023 (55.7)	160,446 (44.3)
	1	241,166 (24.2)	134,245 (55.7)	106,921 (44.3)
	2	140,558 (14.1)	81,174 (57.8)	59,384 (42.2)
	3	89,258 (8.9)	51,827 (58.1)	37,431 (41.9)
	4+	159,417 (16.0)	90,896 (57.0)	68,521 (43.0)
**Zip code demographics (Census 2019)**				
	% White (mean %)	73.8 (76)	73.4% (N/A^a^)	74.3% (N/A)
	% Black (mean %)	11.9 (13)	12.3% (N/A)	11.4% (N/A)
	% Asian (mean %)	5.9 (5)	5.8% (N/A)	6.0% (N/A)
	Median income (mean)	$66,829 ($62,843)	$66,431 (N/A)	$67,343 (N/A)

^a^N/A: not available.

### Overall Model Performance

All models outperformed the logistic regression baseline and were well specified with AUROCs of 0.83 for the THS, 0.89 for heart, 0.86 for lung, 0.84 for neuro, 0.90 for kidney, and 0.83 for digestive functions (Figure 1). All six models were well calibrated. Additional metrics (sensitivity and specificity) can be found in Table 2.

**Figure 2:**
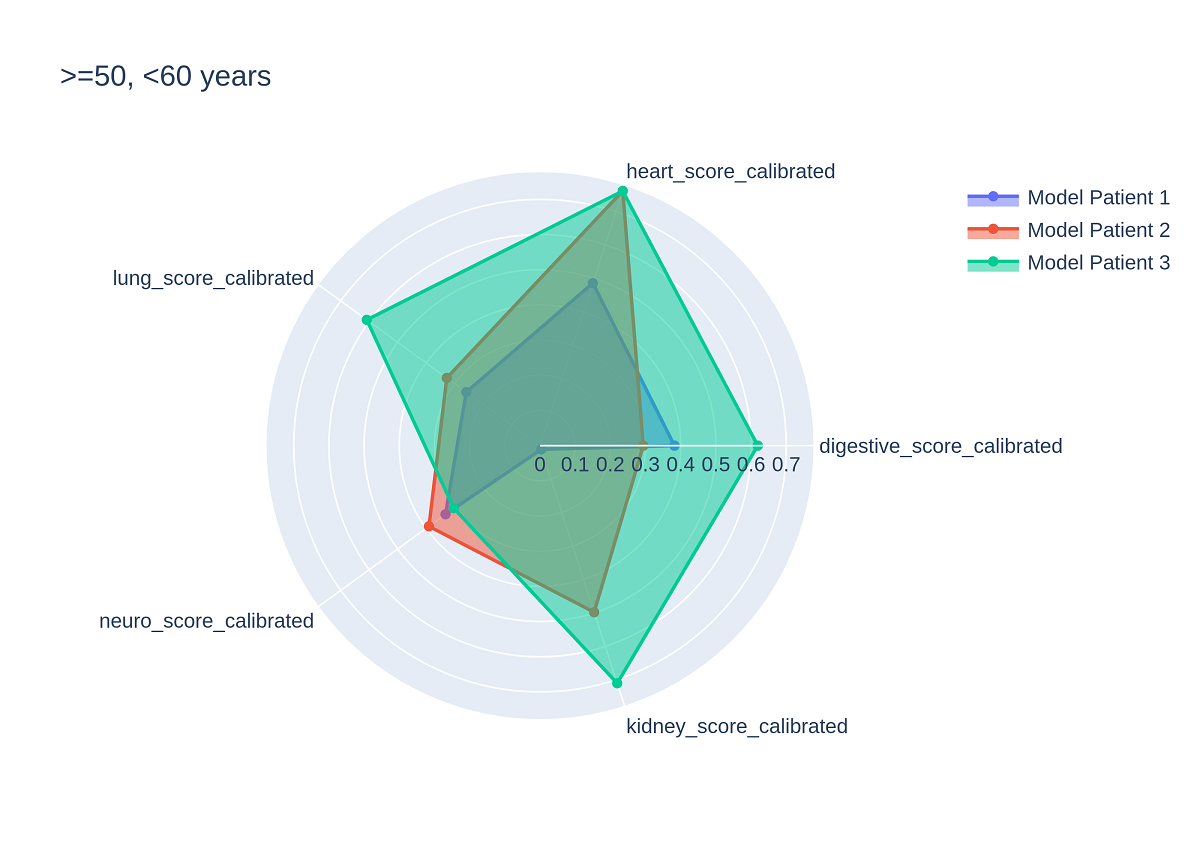
Three patients, all 50-60 years of age and all with approximately equal THSs. THS: total health score.

One benefit of the THP is that it is personalized to the patient to allow for nuanced interpretation based on the affected organ system, in addition to robust predictive performance. Figure 2 demonstrates an illustrative radial plot example of three patients who were around the same age (50-60 years old) and had the same rough THS (>0.8). Unlike grouped scoring systems, the THP enables the clinician to understand the personalized drivers for that score, thereby enabling clinical decisions that are specific to the individual patient. The score of patient 1 was driven primarily by neuro and heart issues, while the score of patient 2 was affected by kidney, neuro, and heart diseases, and the score of patient 3 was mostly affected by heart, lung, digestive, and kidney maladies.

**Table 2:** Gradient-boosted tree AUROC^a^, sensitivity, and specificity for each score in the testing set (n=198,574).

Score type	AUROC (95% CI)	Sensitivity (95% CI)	Specificity (95% CI)
Heart	0.883 (0.876-0.893)	0.82 (0.796-0.845)	0.788 (0.783-0.793)
Lung	0.853 (0.837-0.867)	0.75 (0.713-0.784)	0.802 (0.796-0.808)
Neuro	0.837 (0.821-0.855)	0.756 (0.722-0.793)	0.774 (0.768-0.78)
Kidney	0.892 (0.873-0.908)	0.784 (0.738-0.825)	0.83 (0.824-0.835)
Digestive	0.827 (0.81-0.847)	0.733 (0.698-0.767)	0.756 (0.75-0.762)
THS^b^	0.823 (0.811-0.834)	0.721 (0.701-0.744)	0.777 (0.771-0.783)

^a^AUROC: area under the receiver operating curve.

^b^THS: total health score.

### Important Model Features

**Figure 3:**
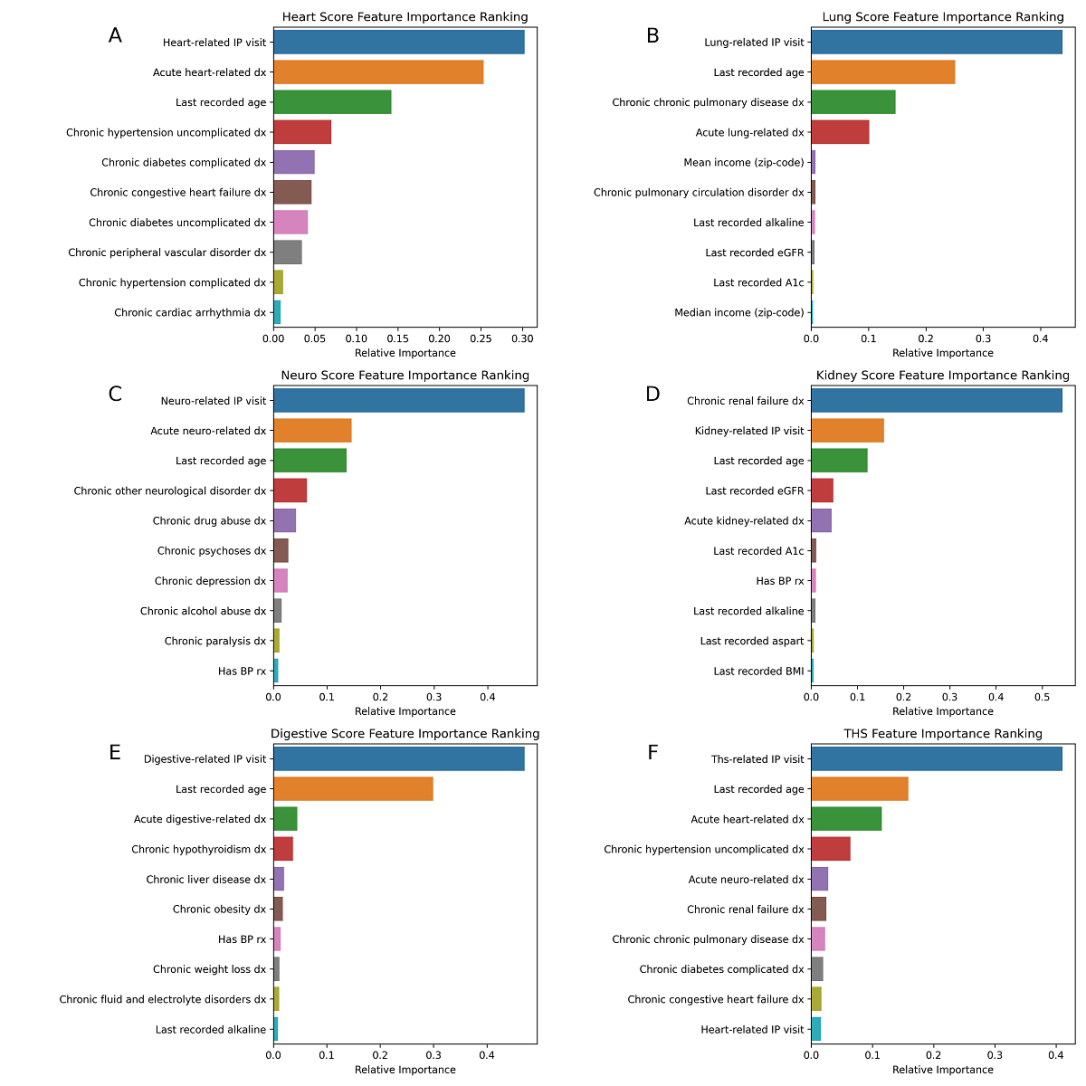
Feature importance plots across all scoring models, generated using Gini impurity reduction. BMI: body mass index; BP, blood pressure; dx: diagnosis; eGFR: estimated glomerular filtration rate; HbA_1c_: hemoglobin A_1c_; IP: inpatient; THS: total health score.

We obtained the most important features of the THS and of each component model. For the THS, the biggest drivers of the model were the use of prior IP hospital visits (0.41) and age (0.15), followed by an acute heart-related diagnosis (0.11), uncomplicated hypertension (0.06), and acute neurological conditions (0.02); see Figure 3. The key features for each component model were directly relevant elements. For example, the most important features of the kidney model included a diagnosis of renal failure, as well as age, last recorded measurement of estimated glomerular filtration rate (eGFR) and hemoglobin A_1c_ (HbA_1c_), and any acute kidney-related diagnosis. Generally, age and a prior history of hospitalization for issues relating to the organ system in question were the most important features across most component models.

### Generalizability Across Subgroups

**Figure 4:**
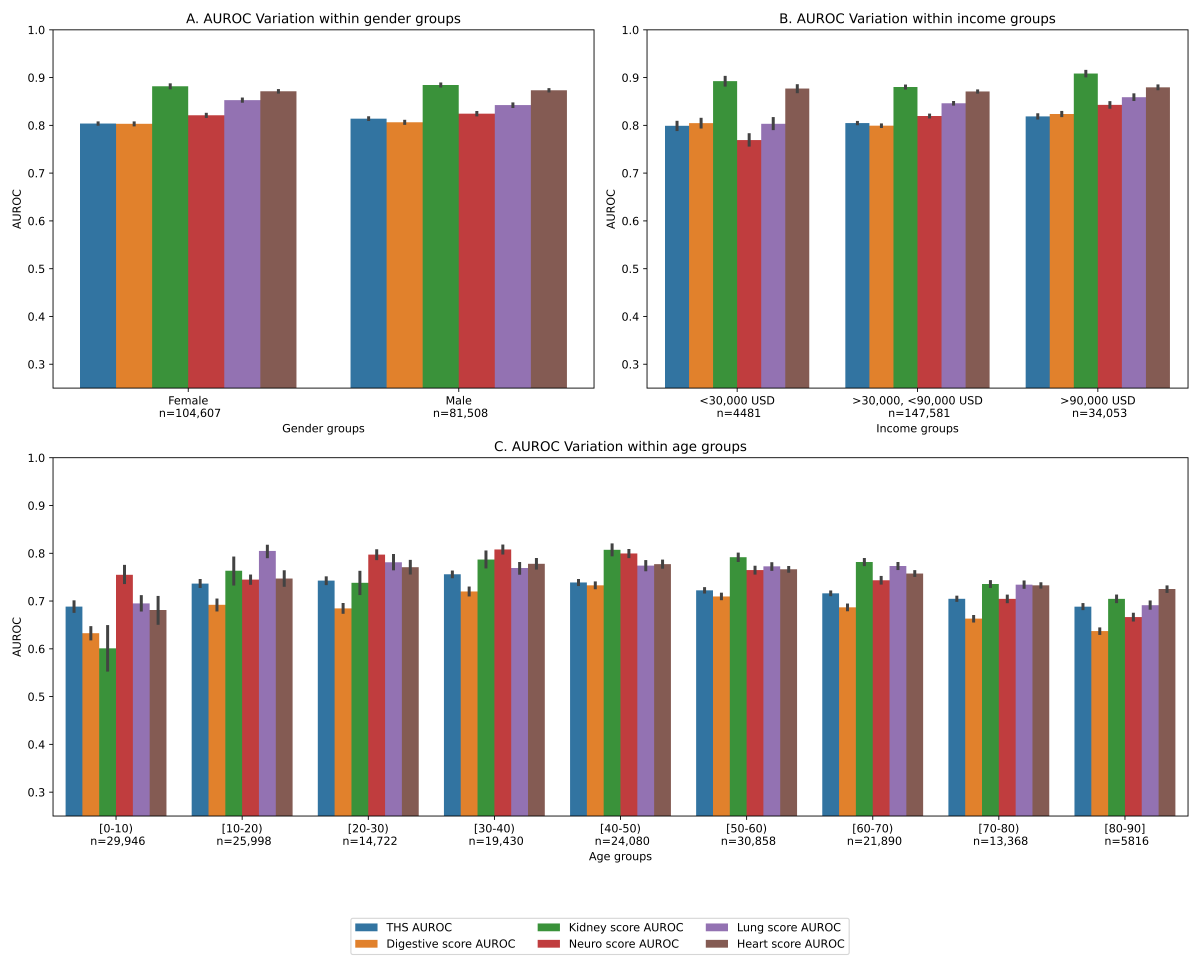
Generalizability. AUROC performance across population subgroups. AUROC: area under the receiver operating curve; THS: total health score.

We examined differences in performance for the THS as well as each component score for population subgroups based on sex, median neighborhood income, and age (Figures 4A, 4B, and 4C, respectively). Statistical AUROC comparisons [24] with Bonferroni corrections revealed that there were no significant differences in model performance on the basis of sex or income. There were occasional statistical differences in performance on the basis of age, primarily related to the 80-90-year-old population, which had significantly fewer data points than any of the other age groups (see Table 1).

### Ablation Tests

We conducted ablation tests of several feature groups to assess the need for them in the final set of models. All ablation test models used procedural data as input, as we assumed access to this information is a given due to the outcome prediction being procedural as well. The combined feature set outperformed the lab, SDoH, and prescription-only models. The combined feature set also outperformed the diagnosis-only model, though not significantly. This statistically nonsignificant outperformance was similarly observed in multiple patient subgroups focused on at-risk patients.

### Performance Comparisons to Charlson and Elixhauser Risk Scores

In addition to a model baseline (logistic regression), we also compared the performance of the THS to commonly used scores of a similar nature, specifically the CCI and the ECI. Both the CCI and the ECI generate a risk score based on different weights associated with certain diseases based on ICD-10 codes. The THS (AUROC=0.82) outperformed both the CCI (AUROC=0.74) and the ECI (AUROC=0.65); see Table 3. We further compared performance across subgroups. Of the baselines, the CCI is a consistently better predictor than the ECI. Across subgroups, the THS also consistently outperformed the score baselines for every age, sex, and income bracket. The improvement was perhaps most pronounced for ages between 20 and 50, as well as for individuals living in lower-income neighborhoods.

**Table 3:** AUROC^a^ performance by sociodemographic strata and score in predicting IP^b^ visitations.

Strata		ECI^c^ score	CCI^d^ score	THS^e^ feature score
All		0.649	0.735	0.823
**Gender^f^**				
	0	0.637	0.733	0.822
	1	0.664	0.736	0.824
**Age bracket**				
	0-10 years	0.621	0.622	0.696
	20-30 years	0.484	0.573	0.725
	20-30 years	0.460	0.621	0.786
	30-40 years	0.509	0.635	0.768
	40-50 years	0.549	0.675	0.769
	50-60 years	0.602	0.693	0.754
	60-70 years	0.637	0.694	0.742
	70-80 years	0.645	0.678	0.721
	80+ years	0.652	0.657	0.711
**Income, median**				
	$0-$30,000	0.640	0.715	0.820
	$30,000-$90,000	0.647	0.734	0.823
	$90,000+	0.665	0.738	0.833

^a^AUROC: area under the receiver operating curve.

^b^IP: inpatient.

^c^CI: Elixhauser Comorbidity Index.

^d^CCI: Charlson Comorbidity Index.

^e^THS: total health score.

^f^0: male; 1: female.

## Discussion

### Principal Findings

There is a continued need for an updated clinical score that profiles patients based on multimorbidities that are equitable across populations and nuanced enough to facilitate precision medicine. To facilitate clinical decision making across patient populations, we created an automated, generalizable, integrated, multimorbidity risk profile across several clinical domains. The THP is composed of cardiovascular, respiratory, neuropsychiatric, renal, and gastrointestinal clinical risk subprofiles, as well as a sixth score, the THS, representing the overall combinatorial risk of the five organ-specific scores. We followed ML best practices to train six integrated models on large-scale EHR data with the forecasted probability of a risk endpoint, organ-specific IP hospital visits, over a 2-year window as the target. We chose IP hospital stays as our risk endpoint because reductions in overall health, whether due to multiple health conditions [6,25] or aging [26], are associated with increased hospital visits [27,28]. The primary contribution of this work goes beyond the models themselves by matching clinical knowledge to data that are available at scale, across a diverse cohort of patients. In our experiments, we found that the profile demonstrates high performance in terms of the AUROC on the aggregate held-out testing set. Importantly, there was consistent performance of this scoring system across sexes, diverse patient ages, and income levels. The THS model and each of the component models learned to generate predictions by focusing on appropriate, clinically relevant patient features. The THP is personalized based on individual organ system risk drivers, and visualizations, such as radar plots, can be used to facilitate explainability and encourage confidence of clinical decision making, providing meaningful feature importance. The THS outperformed relevant baselines, specifically the other commonly used multimorbidity scoring systems CCI and ECI, for every age, sex, and income bracket. Finally, we conducted multiple ablation tests, while retaining procedure features, to determine the relative contribution of feature groups to the THP. In this experiment, we found that while the combined feature set predictive performance outperformed the prescription, lab, and SDoH ablations, it was largely similar to the diagnosis ablation. However, we hypothesized that we would find larger differences in performance among at-risk populations and found, in patients with multiple comorbidities and on certain prescriptions, minor but consistent increases in predictive power using the combined feature set versus the diagnosis feature set, implying that risk prediction is improved on more complex patients, given more complex data. As more features, including more labs, diagnosis, and prescriptions, are added to the THP, future work will more closely examine which demographics benefit from it.

The THP’s multimorbidity scores can be distinguished from traditional multimorbidity scores in three ways: First, they are derived from a comprehensive set of diagnostic, prescription, lab, and medical procedure data. This is in contrast to other multimorbidity scores that use only one set of information, such as diagnoses (as is the case with the ECI [10] and the CCI [9]) or prescriptions (such as Rx-Risk [29]). Second, these scores were derived from a large and diverse cohort of 794,294 patients with medical data spanning decades. Third, the THP was calculated from patients of both sexes and from across the age spectrum (3-90 years), rather than focusing on mostly geriatric populations as with traditional multimorbidity. To the best of our knowledge, this is the first time that ML was used to integrate multiple types of physician-curated clinical information from a large, diverse population and produce a multimorbidity score that can help guide patient care irrespective of sex and age.

As part of the overall multimorbidity score in the THP, we calculated robust, organ-system-specific scores that provide a more granular picture of health. We believe that these organ-specific multimorbidity scores can complement existing condition-specific scores in clinical practice by providing additional validation for treatment decisions for cardiovascular, respiratory, neuropsychiatric, gastrointestinal, and renal domains. Along these lines, we note that these disease-specific scores often use patient-reported outcomes as part of their input [14], with some even using them exclusively [3]. Although EHR software systems may have health-based modules to automatically compute such scores at the population level, these self-reported data are frequently unavailable or unreliable [30], making it difficult to scale these scores to the population level with a high degree of efficacy. Although the THP cannot be directly compared against these alternative risk scores, as they typically focus on diagnoses versus emergency events, the fact that the THP consistently achieved relatively high AUROCs is nevertheless promising with regard to its ability to complement these more specific risk scores. Specifically, it says something well established in multimorbidity scores but understudied in more specific risk scores: foregoing patient input (which typically contains useful information) entirely, in exchange for more scalable data, can still lead to strong results. Moreover, these alternate risk scores are also typically hyperspecific, limiting their clinical utility to a subset of patients—likely due to them being built on similarly restricted cohorts (eg the American Heart Association pooled cohort equations for atherosclerotic cardiovascular disease derived from cohorts exclusively in the age range of 40-79 years). As our approach has no constraints upon individual patients’ age or sex, and are built using a similarly diverse cohort, risk profiles that are applicable to a far larger population can be easily derived. Of course, we assessed generalizability only among three well-known dimensions (age, sex, and income), and there are far more subtle biases that have been observed even among established risk scores, such as the CHADS2VASC stroke score underestimating risk in patients with chronic renal disease [31]. Further study will be needed to fully examine these sorts of biases in our proposed risk models, but even in this case, the scalability of our approach will only make this research simpler to perform.

### Limitations

Data-related limitations of this study include unmeasured variables and incomplete observations. Regarding the former, in this study, we did not include lifestyle behavioral data, such as nutrition, smoking, and physical activity. Although reporting of these factors is known to be inconsistent and unreliable [32], especially in healthy populations (which typically lack recent EHR/claims medical history), they play an important role in clinical outcomes. We believe this would be most addressable through the collection of passive data from wearable sensors, which future work will include. On a similar note, although we were able to use aggregate statistics for race and economic status based on zip-code-derived census data, we were unable to track them at an individual level. Though this form of zip code aggregation has been shown to be useful in clinical risk assessment [33], individual SDoH data could increase the precision and accuracy of THP multimorbidity scores. Future studies of the THP will examine the impact of longer observation and follow-up windows on strategies for clinical intervention. Finally, we note the unreliability of claims data at large, as they are typically produced with financial incentives that are not necessarily aligned with patient care, though they are still often used for risk assessment problems [34,35].

### Conclusion

In summary, we combined practical clinical knowledge with modern ML on large-scale data to produce THP multimorbidity scores to aid in decision making across generalizable patient populations. We believe that the THP will allow for more targeted prioritization of care-gap closure, the assessment of comprehensive risk profiles for a greater number of patients, and facilitation of better physician-patient interactions and joint decision making via feature explainability. Although prospective studies will be required to measure the utility of this approach, our intention is that the THS may be used as a preliminary risk stratifier to rapidly prioritize patients for care from a population health management perspective [36]. Once a patient is engaged with a care provider, the organ-specific scores can be used to guide, and explain, individualized clinical interventions based on existing best practices. This would provide the foundation for an integrated continuum between population health and personalized medicine. Finally, we also note the promise that the THP has for clinical research at large, reflecting the rare opportunity to study holistic clinical risk at an extreme scale, potentially unveiling clinically valuable insights.

## Abbreviations

AHRQ: Agency for Health care Research and Quality
AUC: area under the curve
AUROC: area under the receiver operating curve
CCI: Charlson Comorbidity Index
CPT: Current Procedural Terminology
dx: diagnosis
ECI: Elixhauser Comorbidity Index
eGFR: estimated glomerular filtration rate
EHR: electronic health record
GPI: General Product Identifier
HbA_1c_: hemoglobin A_1c_
HCUP: Healthcare Cost and Utilization Project
HIPAA: Health Insurance Portability and Accountability Act of 1996
ICD-10: International Classification of Diseases, Tenth Revision
IP: inpatient
MI-CLAIM: Minimum Information about Clinical Artificial Intelligence Modeling
ML: machine learning
SDoH: social determinants of health
THP: total health profile
THS: total health score

## References

[1] Huntley AL, Johnson R, Purdy S, Valderas JM, Salisbury C (2012). Measures of multimorbidity and morbidity burden for use in primary care and community settings: a systematic review and guide. Ann Fam Med.

[2] Soh CH, Ul Hassan SW, Sacre J, Maier AB (2020). Morbidity measures predicting mortality in inpatients: a systematic review. J Am Med Dir Assoc.

[3] Newman AB, Boudreau RM, Naydeck BL, Fried LF, Harris TB (2008). A physiologic index of comorbidity: relationship to mortality and disability. J Gerontol A Biol Sci Med Sci.

[4] Fortin M, Bravo G, Hudon C, Lapointe L, Almirall J, Dubois M, Vanasse A (2006). Relationship between multimorbidity and health-related quality of life of patients in primary care. Qual Life Res.

[5] Librero J, Peiró S, Ordiñana R (1999). Chronic comorbidity and outcomes of hospital care. J Clin Epidemiol.

[6] Wolff JL, Starfield B, Anderson G (2002). Prevalence, expenditures, and complications of multiple chronic conditions in the elderly. Arch Intern Med.

[7] Marengoni A, von Strauss E, Rizzuto D, Winblad B, Fratiglioni L (2009). The impact of chronic multimorbidity and disability on functional decline and survival in elderly persons. A community-based, longitudinal study. J Intern Med.

[8] Payne RA, Mendonca SC, Elliott MN, Saunders CL, Edwards DA, Marshall M, Roland M (2020). Development and validation of the Cambridge Multimorbidity Score. CMAJ.

[9] Charlson ME, Pompei P, Ales KL, MacKenzie CR (1987). A new method of classifying prognostic comorbidity in longitudinal studies: development and validation. J Chronic Dis.

[10] van Walraven C, Austin PC, Jennings A, Quan H, Forster AJ (2009). A modification of the Elixhauser comorbidity measures into a point system for hospital death using administrative data. Med Care.

[11] Stirland LE, González-Saavedra Laura, Mullin DS, Ritchie CW, Muniz-Terrera G, Russ TC (2020). Measuring multimorbidity beyond counting diseases: systematic review of community and population studies and guide to index choice. BMJ.

[12] King DE, Xiang J, Pilkerton CS (2018). Multimorbidity trends in United States Adults, 1988-2014. J Am Board Fam Med.

[13] Cassell A, Edwards D, Harshfield A, Rhodes K, Brimicombe J, Payne R, Griffin S (2018). The epidemiology of multimorbidity in primary care: a retrospective cohort study. Br J Gen Pract.

[14] Wilson PW, D'Agostino RB, Levy D, Belanger AM, Silbershatz H, Kannel WB (1998). Prediction of coronary heart disease using risk factor categories. Circulation.

[15] Marengoni A, Onder G (2015). Guidelines, polypharmacy, and drug-drug interactions in patients with multimorbidity. BMJ.

[16] Salisbury C (2013). Multimorbidity: time for action rather than words. Br J Gen Pract.

[17] Alzoubi H, Alzubi R, Ramzan N, West D, Al-Hadhrami T, Alazab M (2019). A review of automatic phenotyping approaches using electronic health records. Electronics.

[18] Pathak J, Kho AN, Denny JC (2013). Electronic health records-driven phenotyping: challenges, recent advances, and perspectives. J Am Med Inform Assoc.

[19] Hassaine A, Salimi-Khorshidi G, Canoy D, Rahimi K (2020). Untangling the complexity of multimorbidity with machine learning. Mech Ageing Dev.

[20] Xu J, Murphy S, Kochanek K, Arias E Mortality in the United States, 2018: NCHS data brief No. 355, January 2020.

[21] Sanchez-Pinto LN, Venable LR, Fahrenbach J, Churpek MM (2018). Comparison of variable selection methods for clinical predictive modeling. Int J Med Inform.

[22] U.S. Preventive Services Task Force Recommendations search results.

[23] Norgeot B, Quer G, Beaulieu-Jones B, Torkamani A, Dias R, Gianfrancesco M, Arnaout R, Kohane I, Saria S, Topol E, Obermeyer Z, Yu B, Butte A (2020). Minimum information about clinical artificial intelligence modeling: the MI-CLAIM checklist. Nat Med.

[24] Hanley JA, McNeil BJ (1982). The meaning and use of the area under a receiver operating characteristic (ROC) curve. Radiology.

[25] Jia H, Lubetkin EI (2016). Impact of nine chronic conditions for US adults aged 65 years and older: an application of a hybrid estimator of quality-adjusted life years throughout remainder of lifetime. Qual Life Res.

[26] Ryan A, Wallace E, O'Hara P, Smith SM (2015). Multimorbidity and functional decline in community-dwelling adults: a systematic review. Health Qual Life Outcomes.

[27] McPhail SM (2016). Multimorbidity in chronic disease: impact on health care resources and costs. Risk Manag Healthc Policy.

[28] Schneider KM, O'Donnell BE, Dean D (2009). Prevalence of multiple chronic conditions in the United States' Medicare population. Health Qual Life Outcomes.

[29] Von Korff M, Wagner EH, Saunders K (1992). A chronic disease score from automated pharmacy data. J Clin Epidemiol.

[30] Prince SA, Adamo KB, Hamel ME, Hardt J, Connor Gorber S, Tremblay M (2008). A comparison of direct versus self-report measures for assessing physical activity in adults: a systematic review. Int J Behav Nutr Phys Act.

[31] Foote C, Woodward M, Jardine MJ (2017). Scoring risk scores: considerations before incorporating clinical risk prediction tools into your practice. Am J Kidney Dis.

[32] Coughlin SS (1990). Recall bias in epidemiologic studies. J Clin Epidemiol.

[33] Kasthurirathne SN, Vest JR, Menachemi N, Halverson PK, Grannis SJ (2018). Assessing the capacity of social determinants of health data to augment predictive models identifying patients in need of wraparound social services. J Am Med Inform Assoc.

[34] Patterson-Lomba O, Ayyagari R, Carroll B (2019). Risk assessment and prediction of TD incidence in psychiatric patients taking concomitant antipsychotics: a retrospective data analysis. BMC Neurol.

[35] Martin J, Mills S, Foley ME (2018). Innovative models of dental care delivery and coverage: patient-centric dental benefits based on digital oral health risk assessment. Dent Clin North Am.

[36] Haas LR, Takahashi PY, Shah ND, Stroebel RJ, Bernard ME, Finnie DM, Naessens JM (2013). Risk-stratification methods for identifying patients for care coordination. Am J Manag Care.

